# Investigating the In Vivo Effects of Anti-Prion Protein Nanobodies on Prion Disease with AAV Vector

**DOI:** 10.3390/pathogens14020131

**Published:** 2025-02-02

**Authors:** Jingjing Zhang, Mengfei Wang, Dan Wang, Xiangyi Zhang, Yue Ma, Els Pardon, Jan Steyaert, Romany Abskharon, Fei Wang, Jiyan Ma

**Affiliations:** 1 School of Basic Medical Sciences, Capital Medical University, Beijing 100069, China; 2 Beijing Institute for Brain Research, Chinese Academy of Medical Sciences & Peking Union Medical College, Beijing 102206, China; 3 Chinese Institute for Brain Research, Beijing 102206, China; 4 Department of Neurodegenerative Science, Van Andel Institute, Grand Rapids, MI 49503, USA; 5 VIB-VUB Center for Structural Biology, VIB, Pleinlaan 2, 1050 Brussels, Belgiumrnn2222@yahoo.com (R.A.); 6 Structural Biology Brussels, Vrije Universiteit Brussel (VUB), Pleinlaan 2, 1050 Brussels, Belgium; 7 National Institute of Oceanography and Fisheries (NIOF), Cairo 11516, Egypt; 8 Department of Neurology, McGovern Medical School, The University of Texas Health Science Center at Houston, Houston, TX 77030, USA

**Keywords:** prion, transmissible spongiform encephalopathy, nanobody, therapy, adeno-associated virus

## Abstract

Prion diseases are fatal neurodegenerative disorders affecting humans and animals, and the central pathogenic event is the conversion of normal prion protein (PrP^C^) into the pathogenic PrP^Sc^ isoform. Previous studies have identified nanobodies that specifically recognize PrP^C^ and inhibit the PrP^C^ to PrP^Sc^ conversion in vitro. In this study, we investigated the potential for in vivo expression of anti-PrP^C^ nanobodies and evaluated their impact on prion disease. The coding sequences of three nanobodies were packaged into recombinant adeno-associated virus (rAAV) and were administered via intracerebroventricular (ICV) injection in newborn mice. We found that the expression of these nanobodies remained robust for over 180 days, with no observed detrimental effects. To assess their therapeutic potential, we performed ICV injections of nanobody-expressing rAAVs in newborn mice, followed by intracerebral prion inoculation at 5–6 weeks of age. One nanobody exhibited a small yet statistically significant therapeutic effect, extending survival time from 176 days to 184 days. Analyses of diseased brains revealed that the nanobodies did not alter the pathological changes. Our findings suggest that high levels of anti-PrP^C^ nanobodies are necessary to delay disease progression. Further optimization of the nanobodies, AAV vectors, or delivery methods is essential to achieve a significant therapeutic effect.

## 1. Introduction

Prion disease, also known as transmissible spongiform encephalopathies (TSEs), is a group of fatal neurodegenerative disorders, including Creutzfeldt–Jakob disease and Gerstmann–Straussler–Scheinker disease in humans and bovine spongiform encephalopathy and chronic wasting disease in animals [1,2]. The central pathogenic event in prion disease is the conformational conversion of host-encoded PrP^C^ into the pathogenic PrP^Sc^ isoform [3]. Due to its self-perpetuating nature, PrP^Sc^ can seed the conversion of PrP^C^ to PrP^Sc^, leading to the increased accumulation of PrP^Sc^ [4]. PrP^Sc^ alters the endogenous PrP^C^, and once the level of aberrant PrP^C^ exceeds a toxic threshold, rapid neurodegeneration ensues [5,6]. Consequently, the disease typically exhibits a prolonged incubation period; however, once clinical symptoms appear, it progresses rapidly and often culminates in death within a short timeframe. The neuropathology of the disease is characterized by spongiform vacuolation (spongiosis), aberrant PrP deposition, and gliosis [7]. Currently, these diseases remain invariably incurable, with no available treatments [1,2]. 

Significant efforts have been made to develop therapies for prion diseases [8,9,10]. Given that the conformational conversion of PrP is the critical pathogenic step [3], plausible therapeutic strategies include targeting the pathogenic PrP^Sc^ to reduce its seeding activity or targeting the normal PrP^C^ to prevent its conversion. Small molecules that can penetrate the blood–brain barrier (BBB) and act against PrP^Sc^ would be the preferred choice; however, this approach encounters challenges due to the presence of prion strains, which arise from minor structural differences in PrP^Sc^ molecules [11]. Small molecules may be effective against certain prion strains, but ineffective against others [12].

Immunotherapy is another promising strategy for prion treatment, attributed to its strong specificity and relatively few side effects [9]. Generally, antibody–antigen interactions are more tolerant of small structural variations [13]. A vaccination approach is likely to elicit a polyclonal response that is capable of accommodating more structural variations in prions. Passive immunotherapy using antibodies against PrP^C^ is another viable approach. Given that PrP^C^ is the obligate substrate for all prion strains, antibody binding to PrP^C^ to prevent its conformational conversion could potentially inhibit the propagation of all prion strains. Among the antibodies tested, the ICSM18 antibody has emerged as the most effective, extending the average survival time of mice intraperitoneally inoculated with RML-prions to over 500 days [14]. PRN100, a humanized version of ICSM18, has been trialed in six patients, showing a favorable safety profile and some encouraging findings [15]. However, limitations of anti-PrP^C^ treatment include its general ineffectiveness when initiated at the late stage of the disease [9], the potential for certain anti-PrP^C^ antibodies to induce acute neurotoxicity [16,17], and the challenges associated with antibodies crossing the blood–brain barrier [18]. 

In addition to conventional antibodies, nanobodies have garnered increased attention in biomedical research. Nanobodies (Nbs) are the antigen-binding domains of single heavy-chain antibodies that naturally occur in sharks and camelids [19]. Unlike conventional antibodies, which possess a complex structure comprising heavy and light chains with a larger molecular weight (~150 kDa), Nbs are typically smaller with a molecular weight of approximately 12–14 kDa [19]. They can exhibit high affinities for their cognate antigens, demonstrate remarkable stability, and can be conveniently produced as recombinant proteins. Furthermore, it has been reported that some Nbs are capable of crossing the BBB [20], presumably due to their smaller size and tightly packed structure. 

Nbs have been extensively utilized across various fields, including, but not limited to, tumors, neurodegenerative disorders, infectious diseases, and inflammation, as well as for molecular imaging and tracking [21]. It has been shown that Nbs targeting alpha-synuclein for proteasome degradation mitigate disease phenotypes in a Parkinson’s disease model [22]. Marino et al. have revealed that anti-BACE1 Nb can produce long-term positive effects on amyloid load, neuroinflammation, synaptic function, and cognitive performance in an Alzheimer’s disease mouse model [23]. 

In prion research, several Nbs have been identified that specifically recognize PrP^C^ and inhibit prion propagation in the biochemical assay and in prion-infected cultured cells [24,25]. However, their in vivo effects remain unknown. We investigated the potential for expressing these anti-PrP^C^ Nbs in the central nervous system (CNS) and assessed whether they induce acute neurotoxicity in vivo and influence the progression of prion disease.

## 2. Materials and Methods

### 2.1. Plasmids

All plasmids were maintained and amplified in Trans5α Chemically Competent Cell (cat#CD201-01, TransGen, Beijing, China). Constructs were created using standard molecular cloning techniques. A 3 × FLAG tag (DYKDHDGDYKDHDIDYKDDDDK) was inserted immediately in the C-terminal of the Nb coding sequences. The DNA fragments were amplified using PCR with the following primers: Nb against GFP (Nb(GFP)): forward primer: 5′-GGCCTCTGCAAAAAGCGGGTCCAACTGGTGGAGTCTG-3′; reverse primer: 5′-CACCGTCATGGTCTTTGTAGTCGCTGGAGACGGTGACCTGGGTG-3′; 5′-CCTTGTAATCGATGTCATGATCTTTATAATCACCGTCATGGTCTTTG-3′; 5′-GATATCGAATTCTTACTTGTCATCGTCATCCTTGTAATCGATGTCATG-3′. Nb196 against PrP^C^: forward primer: 5′-GTCGGCCTCTGCAAAAAGCGGGCTAGCCAGGTGCAGCTGCAGGAG-3′; reverse primer: 5′-CACCGTCATGGTCTTTGTAGTCGTCGACTGAGGAGACGGTGACCTGG-3′; 5′-CCTTGTAATCGATGTCATGATCTTTATAATCACCGTCATGGTCTTTG-3′; 5′-GATATCGAATTCTCACTTGTCATCGTCATCCTTGTAATCGATGTCATG-3′. Nb484 against PrP^C^: forward primer: 5′-GTCGGCCTCTGCAAAAAGCGGGCTAGCCAGGTGCAGCTGCAGGAG-3′; reverse primer: 5′-CACCGTCATGGTCTTTGTAGTCGTCGACTGAGGAGACGGTGACCTGG-3′; 5′-CCTTGTAATCGATGTCATGATCTTTATAATCACCGTCATGGTCTTTG-3′; 5′-GATATCGAATTCTCACTTGTCATCGTCATCCTTGTAATCGATGTCATG-3′. Nb862 against PrP^C^: forward primer: 5′-GTCGGCCTCTGCAAAAAGCGGGCTAGCCAGGTGCAGCTGCAGG-3′; reverse primer: 5′-CACCGTCATGGTCTTTGTAGTCGTCGACTGAGGAGACGGTGACCTGG-3′; 5′-CCTTGTAATCGATGTCATGATCTTTATAATCACCGTCATGGTCTTTG-3′; 5′-GATATCGAATTCTCACTTGTCATCGTCATCCTTGTAATCGATGTCATG-3′.

A second round of PCR was performed to insert the DNA sequence of the signal peptide of mouse PrP (GenBank accession number M18070) to the N-terminus of the Nb coding sequences, which allows for the Nb to enter the secretory pathway. The following primers were used: Nb(GFP): forward primer: 5′-CATTTTGGCAAAGAATTGGATCCATGGCGAACCTTGGCTACTGGCTGC-3′; reverse primer: 5′-CAGACTCCACCAGTTGGACCCGCTTTTTGCAGAGGCC-3′. Nb196: forward primer: 5′-CATTTTGGCAAAGAATTGGATCCATGGCGAACCTTGGCTACTGGCTGC-3′; reverse primer: 5′-CTCCTGCAGCTGCACCTGGCTAGCCCGCTTTTTGCAGAGGCCGAC-3′. Nb484: forward primer: 5′-CATTTTGGCAAAGAATTGGATCCATGGCGAACCTTGGCTACTGGCTGC-3′; reverse primer: 5′-CTCCTGCAGCTGCACCTGGCTAGCCCGCTTTTTGCAGAGGCCGAC-3′. Nb862: forward primer: 5′-CATTTTGGCAAAGAATTGGATCCATGGCGAACCTTGGCTACTGGCTGC-3′; reverse primer: 5′-CCTGCAGCTGCACCTGGCTAGCCCGCTTTTTGCAGAGGCCGAC-3′.

The second-round PCR products were ligated between Bam HI and Eco RI sites of the vector pAAV-CAG-tdTotmato (provided by the Vector Core at the Chinese Institute for Brain Research, Beijing (CIBR)), yielding plasmids pAAV-CAG-Nb(GFP)-FLAG, pAAV-CAG-Nb196-FLAG, pAAV-CAG-Nb484-FLAG, and pAAV-CAG-Nb862-FLAG.

### 2.2. Expression of Nanobodies in Mice

The plasmids were packaged into rAAV-PHP.eB by the Vector Core at CIBR. Newborn pups were anesthetized on ice and were then subjected to bilateral injection into lateral ventricles, with a total rAAV dosage of 3 × 10^10^ viral genome copies (gc). Mice were euthanized to collect tissues at 30, 90, 150, and 180 days post-injection for enzyme-linked immunosorbent assay (ELISA) and Western blotting (WB) analyses. Following perfusion with PBS, the brain tissues were dissected into the olfactory bulb (OB), cortex (CTX), hippocampus (HIP), midbrain (MB), brainstem (BS), and cerebellum (CERE). Additionally, spinal cords and peripheral tissues—including heart, liver, lung, kidney, muscle, and colon—were also collected and stored at −80 °C.

### 2.3. Prion Inoculation

Five- to six-week-old female mice were anesthetized and the fur on their heads was removed. Following sterilization, prion inoculum was injected into the mouse’s brain using a 1 mL syringe. The injection was performed slowly with 30 μL inoculum per mouse at a vertical depth of 1 mm on the right side of the sagittal suture where the parietal bones meet, and approximately 1 mm anterior to the lambda suture where the sagittal suture meets the occipital bone. The injection duration was maintained for 1 min, and the needle was kept in place for an additional minute after the injection. After the inoculation, mice were closely monitored for signs of prion disease. All mice were weighed monthly for the first five months and daily after 150 days post prion inoculation. Mice were considered to be in the terminal stage when their body weight fell below 18 g. At that point, they were euthanized, and tissues were collected for further analysis. The prion inoculum was 1% brain homogenate prepared from a mouse that succumbed to terminal prion disease induced by the intracerebral inoculation of a recombinant prion [26].

### 2.4. Biochemical Analysis

After homogenization with 1× PBS, the tissue homogenate (10% *w*/*v*) was prepared at 4 °C. For WB analysis, the desired volume of the homogenate was mixed with a 10 × cell lysis buffer (5% Triton X-100 and 5% sodium deoxycholate), and the protein concentration was determined using a BCA assay kit (cat#23227, Thermo Fisher Scientific Inc., Waltham, MA, USA). Following the addition of PMSF to a final concentration of 1 mM (cat#BL507A, Biosharp, Hefei, China), the extracted proteins were mixed with SDS-PAGE loading buffer and incubated for 10 min at 95 °C. A total of 40 μg of protein was loaded for SDS-PAGE, and WB was performed as previously described [27]. The antibodies utilized were mouse anti-FLAG (1:5000, cat#F1804, Sigma-Aldrich, Saint Louis, MO, USA), mouse anti-β-actin (1:2000, cat#A1978, Sigma-Aldrich, Saint Louis, USA), 6D11 mouse anti-PrP (1:2000, cat#808003, BioLegend, Inc., San Diego, CA, USA), 3F10 mouse anti-PrP (1:10,000, a generous gift from Dr YS Kim), and HRP-conjugated Affinipure Goat Anti-Mouse IgG (H+L) (1:5000, cat#SA00001, Proteintech, Rosemont, IL, USA). The analysis was conducted using the iBrightTM analysis software (Thermo Fisher Scientific Inc., Waltham, USA).

For proteinase K (PK) digestion, 40 μg of brain lysates were digested with 30 μg/mL PK at 37 °C for 1 h. The digestion was terminated by adding PMSF to a final concentration of 2 mM, followed by a 5 min incubation on ice. The PK-resistant PrP fragments were then separated by 14% SDS-PAGE and detected via WB analysis using the 6D11 and 3F10 anti-PrP antibody.

For ELISA, 96-well plates (cat#439454, Thermo Fisher Scientific Inc., Waltham, USA) were coated overnight at 4 °C with 2 μg/mL protein lysates in sodium bicarbonate buffer (0.1 M NaHCO_3_, pH 8.0) and subsequently washed with PBST (PBS plus 0.05% Tween). Residual protein binding sites in the wells were blocked with 2% BSA in PBS for two hours at room temperature. The wells were then incubated with mouse anti-FLAG (1:10,000, cat#F1804, Sigma-Aldrich, Saint Louis, USA) at room temperature for 2.5 h and then washed with PBST, followed by HRP-conjugated Affinipure Goat Anti-Mouse IgG (H+L) (1:3000, cat#SA00001, Proteintech, Rosemont, USA) for 1.5 h at room temperature. Finally, 1-step ultra TMB solution (Cat# 34028, Thermo Fisher Scientific Inc., Waltham, USA) was added to each well for 30 min at room temperature, followed by the addition of a TMB stopping solution (2 M H_2_SO_4_) to stop the reaction. Absorbance at 450 nm was measured using a microplate reader (FLUOstar Omega, BMG Labtech, Offenburg, Germany).

### 2.5. Pathological Analysis

After fixation in 10% formalin (cat#G2161, Solarbio, Beijing, China) for 24 h, the brains were immersed in 88% formic acid (cat#F112034, Aladdin, Shanghai, China) for 1.5 h, after which they were fixed again with 10% formalin for an additional 24 h. The tissues were washed with PBS and underwent a gradient ethanol dehydration, followed by xylene processing, before being embedded in paraffin blocks. Hematoxylin-eosin (HE) staining was performed according to the protocol provided by the HE Staining Kit (cat#G1120, Solarbio, Beijing, China), and immunohistochemical staining was conducted following the mouse/rabbit hypersensitive polymer assay system protocol (cat#PV-8000, ZSGB-Bio, Beijing, China). The antibodies utilized included rabbit anti-Iba1 (1:1500, cat#ab178846, Abcam, Cambridge, UK), rabbit anti-GFAP (1:500, cat#bs-0199R, Bioss, Beijing, China), and SAF84 mouse anti-PrP (1:500, cat#189775, CAYMAN, Ann Arbor, MI, USA).

### 2.6. Behavioral Analysis

To evaluate the motor coordination and balance of prion-inoculated mice, we conducted the rotarod test at 150 days post prion inoculation. Initially, mice were placed on the rod at rest or at a slow speed to acclimate to the environment and the task. Following a 24 h rest period, the mice were again placed on the rod at rest for 2 min, during which the time each mouse spent on the rotarod before falling was recorded (maximum of 180 s). The rod speed was subsequently increased to 2 rpm, and the duration of time spent on the rod was also recorded (maximum of 120 s). Each mouse underwent three trials to ensure consistency and reliability.

### 2.7. Statistical Analysis

Protein level analysis and survival curve plotting were conducted using GraphPad Prism 10.1.2 software. All survival curves were analyzed using the Kaplan–Meier log-rank (Mantel–Cox) method. Error bars in the figures represent the standard deviation. An unpaired Student’s *t*-test was used in each experiment as indicated in the figure legends. **** indicates *p* < 0.0001; *** represents *p* < 0.001; ** represents *p* < 0.01; * represents *p* < 0.05.

## 3. Results

### 3.1. Expression of Nanobodies in the CNS

**Figure 1:**
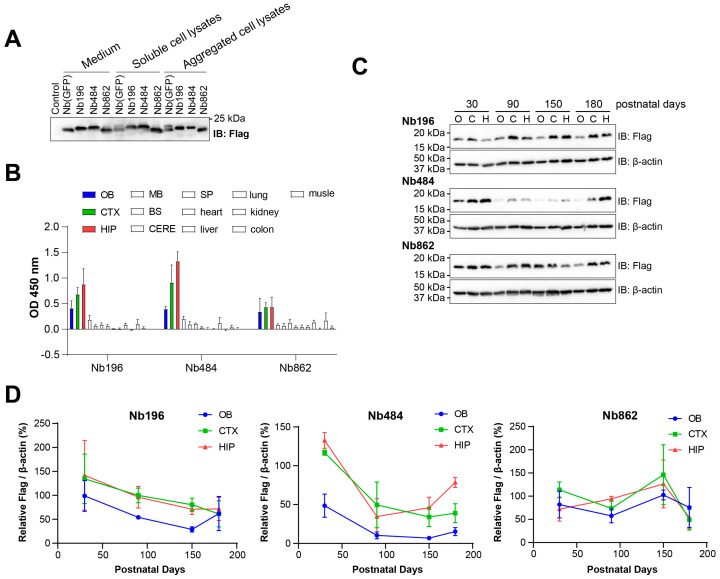
Expression of Nbs in 293T cells and in mice. (**A**). Cultured 293T cells were transiently transfected with plasmids expressing Nb(GFP), Nb196, Nb484, or Nb862. Twenty-four hours post-transfection, medium was replaced with serum-free cell culture medium, and cells were cultured for additional 24 h. The levels of the indicated Nbs in the medium, and the soluble and aggregated fractions of cell lysates were detected using an immunoblot (IB) analysis using an anti-Flag antibody (Flag), with the medium from un-transfected cells serving as a negative control. (**B**). Thirty days following ICV injection of rAAV, the expression levels of the indicated Nbs in the olfactory bulb (OB), cortex (CTX), hippocampus (HIP), midbrain (MB), brainstem (BS), cerebellum (CERE), spinal cord (SP), heart, liver, lung, kidney, colon, and muscle were measured using ELISA. (**C**). The levels of the indicated Nbs in the olfactory bulb (O), cortex (C), and hippocampus (H) of mice at 30, 90, 150, and 180 days post-ICV injection of rAAV were analyzed using immunoblot (IB) analysis using an anti-Flag antibody (Flag). Immunoblot (IB) analysis with an anti-β-actin antibody (β-actin) was performed as the loading control. (**D**). Quantitative analysis of the protein levels as shown in panel c (*n* = 3, normalized to β-actin).

To investigate the potential for expressing anti-PrP^C^ nanobodies in the CNS, we selected three anti-PrP^C^ nanobodies (Nb196, Nb484, and Nb862) which have been previously demonstrated to possess a high binding affinity for normal PrP^C^ [24,25]. Additionally, we included a negative control nanobody directed against GFP, denoted as Nb(GFP) [28]. To determine whether these nanobodies could be effectively expressed, folded, and secreted in mammalian cells, we transiently transfected 293T cells with the corresponding plasmids. After replacing the medium with serum-free medium at 24 h post-transfection, we collected the cell culture medium and lysed the cells at 48 h post-transfection. Cell lysates were separated into soluble and aggregated fractions using centrifugation. A Western blotting (WB) analysis was performed to detect the presence of each Nb in these fractions (Figure 1A). Notably, a sufficient amount of each Nb was detected in the medium, indicating that the expression system functions well in mammalian cells. 

To achieve in vivo expression of these Nbs, we packaged the plasmids into rAAVs, which were subsequently used for ICV injection in newborn mice. We assessed nanobody expression at 30 days post-injection (dpi) using ELISA (Figure 1B), which revealed substantial nanobody expression in the CNS, particularly in the olfactory bulb (OB), cortex (CTX), and hippocampus (HIP), with minimal expression observed in peripheral tissues. To evaluate the duration of Nb expression in the CNS, we collected mouse brains at 30, 90, 150, and 180 dpi and dissected the highest-expressing brain regions. WB analyses indicated that expression levels remained relatively stable, with the exception of Nb484, which showed a significant reduction in all three brain regions (Figure 1C–D).

**Figure 2:**
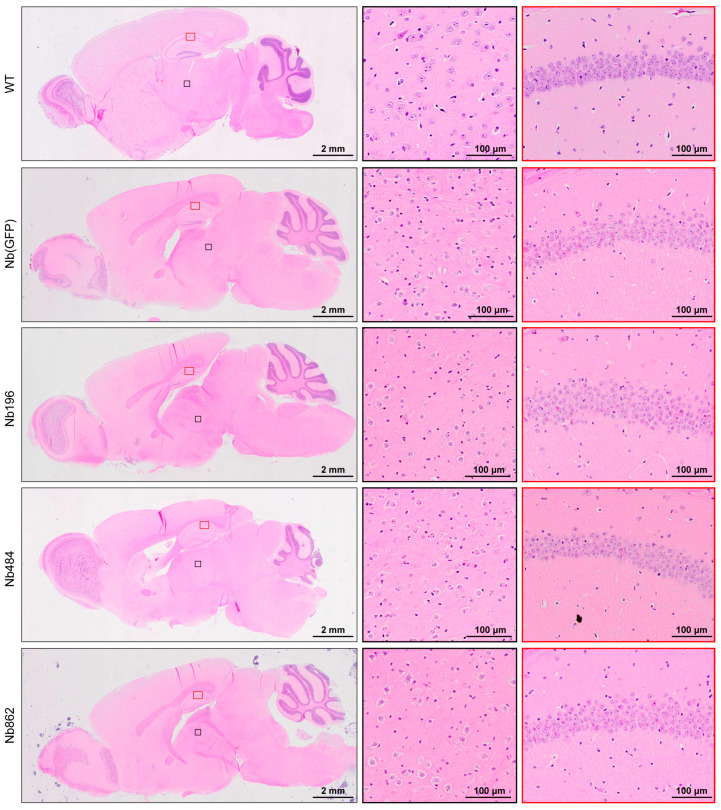
The anti-PrP^C^ nanobodies do not elicit evident neurotoxicity. Representative images of HE staining from mice expressing the indicated Nbs for over 600 days. The brain of a wild-type (WT) mouse without injection serves as a negative control. The middle panels are high-magnification images of the black box in the left panels, and the right panels are high-magnification images of the red box in the left panels. Scale bar for left panels = 2 mm; scale bar for middle and right panels = 100 μm.

Given previous reports indicating that certain anti-PrP^C^ antibodies can induce acute neurotoxicity [16,17], we closely monitored the mice expressing these Nbs for over 600 days. No abnormalities were observed in any of the nanobody-expressing mice. Hematoxylin and eosin (HE) staining also revealed normal brain structures in these mice (Figure 2). Collectively, we conclude that the PrP^C^-binding nanobodies Nb196, Nb484, and Nb862 can be expressed in the CNS at high levels for extended periods, and these nanobodies do not elicit any obvious neurotoxicity.

### 3.2. The Influence of CNS-Expressed Anti-PrP^C^ Nanobodies on Clinical Manifestations of Prion Disease

**Figure 3:**
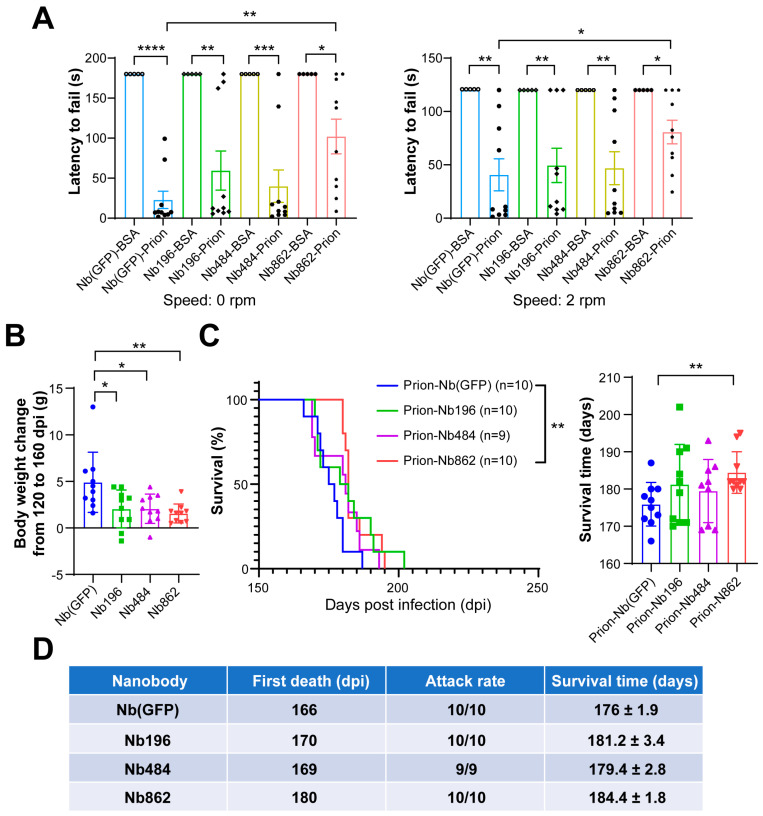
The influence of CNS-expressed Nbs on clinical manifestations of prion disease. (**A**). Performance of mice on the rotarod apparatus at 150 days post prion inoculation. Comparison of latency to fall at speeds of 0 rpm (**left**) or 2 rpm (**right**) in mice expressing Nb(GFP), Nb196, Nb484, or Nb862, which were intracerebrally inoculated with prion or BSA as indicated (n = 10). Statistical analysis was conducted using an unpaired Student’s *t*-test. (**B**). The changes in the bodyweights of the indicated mice from 120 dpi to 160 dpi. Statistical analysis was conducted using one-way ANOVA. (**C**). Survival curves (left) and survival times (right) of mice that received an ICV injection of rAAV-expressing nanobodies followed by intracerebral prion inoculum at 6 weeks of age. (**D**). The time of first death and attack rate of mice that received an ICV injection of rAAV-expressing Nbs followed by intracerebral prion inoculation.

To evaluate the influence of these nanobodies on prion disease, we performed ICV injections of Nb-expressing rAAV in newborn pups, followed by intracerebral prion inoculation at 5–6 weeks of age. At 150 days post prion inoculation, a rotarod test was performed to assess the motor coordination and balance of prion-inoculated mice. Figure 3A shows that all prion-infected mice exhibited motor dysfunction at that time. However, compared to mice expressing the control Nb(GFP) and the other two anti-PrP^C^ Nbs, mice expressing Nb862 performed significantly better in the rotarod test. Additionally, we compared changes in mice bodyweights from 120 dpi, which approximately corresponds to the onset of the clinical phase of prion disease, to 160 dpi, the time point at which nearly all prion-inoculated mice developed clinical signs. Notably, the bodyweight changes observed in mice expressing three anti-PrP^C^ Nbs were significantly lower than those in mice expressing the control Nb(GFP), with Nb 862 exhibiting the most pronounced delay in weight loss. These results suggest that all three Nbs may have an effect on slowing the clinical progression of prion disease (Figure 3B). Consistent with observations made during the clinical phase, the survival time for Nb862-expressing mice was significantly extended from 176 ± 1.9 days to 184 ± 1.8 days (Figure 3C), with the first death of a prion-infected mouse delayed from 166 days to 180 days (Figure 3D). These data indicate that the anti-PrP^C^ Nb862 has a modest yet significant beneficial effect on prion disease.

### 3.3. Nanobodies Did Not Alter the Pathological Changes Associated with Prion Disease

**Figure 4:**
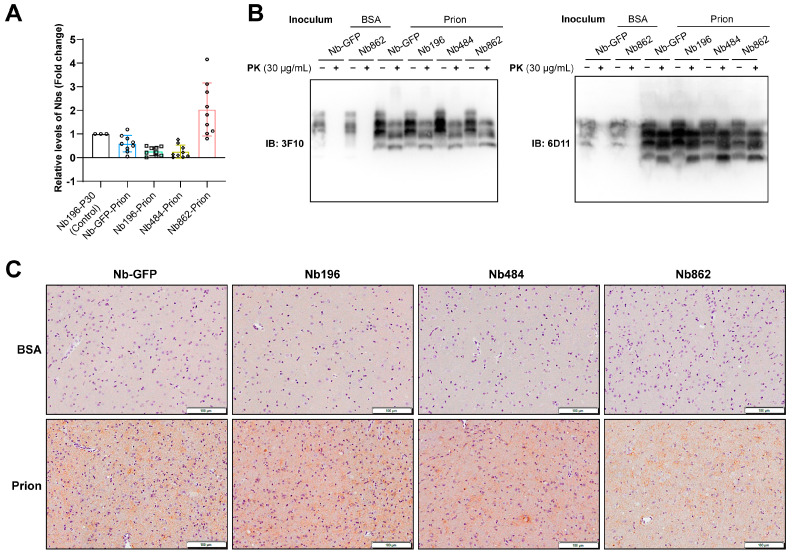
CNS-expressed Nbs did not alter the pathological changes in prion disease. (**A**). Quantitative analysis of the levels of Nbs at the terminal stage of prion disease, as detected by WB (*n* = 10, except for Nb862-Prion *n* = 9, normalized to β-actin). Brain homogenates of mice received ICV injection of rAAV-expressing Nb196 (collected 30 days post-ICV injection) were used as a control for this analysis. (**B**). Brain lysates from the indicated mice were subjected to PK digestion at 37 °C for 1 h at a concentration of 30 μg/mL. PK-resistant PrP was detected using immunoblot (IB) analysis using the 3F10 (**left panel**) and 6D11 (**right panel**) anti-PrP antibodies as indicated. (**C**). Immunohistochemical staining was conducted using SAF84 anti-PrP antibody to determine the pattern of aberrant PrP deposition in Nb-expressing mice received intracerebral inoculation of prion or BSA as indicated (scale bar = 100 μm). All sections were stained at the same time with exactly the same parameters.

**Figure 5:**
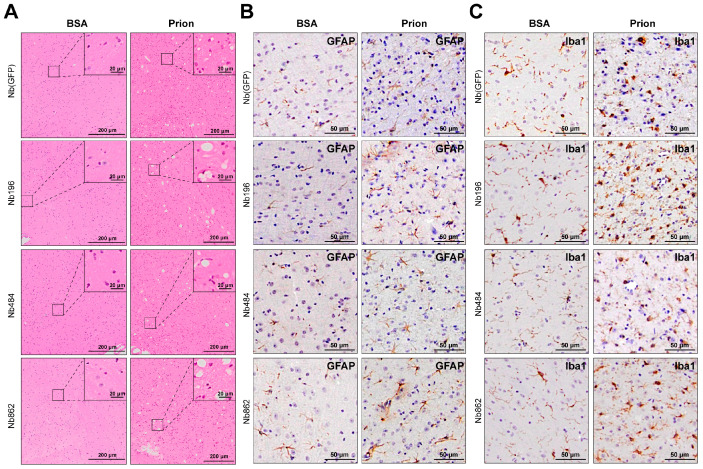
Spongiosis and gliosis were not affected by CNS expression of Nbs. Representative images of histopathological analyses of Nb-expressing mice that received intracerebral inoculation of prion or BSA, as indicated. Brain sections were stained with HE to show the spongiosis (**A**), scale bar = 200 μm), immunohistochemical staining with an anti-GFAP antibody (GFAP) to illustrate astrogliosis (**B**), scale bar = 50 μm), and immunohistochemical staining with an anti-Iba1 antibody (Iba1) to show microgliosis (**C**), scale bar = 50 μm).

Biochemical and pathological analyses were conducted to assess the impact of the Nbs. First, WB analysis was performed to evaluate the levels of Nbs at the terminal stage of prion disease. Figure 4A shows that, compared to the levels of Nb196 at 30 days of age, the levels of Nbs were reduced, with the exception of Nb862, which remained robust. Despite the relatively high levels of Nb862, we did not observe any significant reduction in PK-resistant PrP^Sc^ in Nb862-expressing mice at the terminal stage of prion disease (Figure 4B). Immunohistochemical staining using the SAF84 anti-PrP antibody revealed similar synaptic deposition of aberrant PrP in diseased brains (increased staining intensity compared to BSA-inoculated control samples), which was unaffected by Nb expression (Figure 4C). Additionally, spongiosis and gliosis were also not altered by Nb expression (Figure 5). Overall, we conclude that, in mice succumbing to terminal prion disease, the expression of anti-PrP^C^ Nb at current levels does not modify the pathological changes. 

## 4. Discussion

Our findings reveal that widespread and high-level CNS expression of these three anti-PrP^C^ Nbs does not lead to any detrimental effects in mice, which is consistent with previous findings that utilize cerebellar brain slice cultures [25]. By employing the method of ICV injection of rAAV in newborn mice, we achieved high levels of Nb expression within the CNS; however, only Nb862 exhibited a modest therapeutic effect. 

This limited therapeutic outcome is likely attributable to two factors. First, these Nbs specifically target PrP^C^, which is highly expressed in the CNS [29]. Consequently, higher concentrations of Nbs may be necessary to elicit meaningful therapeutic effects. In addition, although rAAV ICV injection can lead to high levels of transgene expression, this expression appears to be predominantly localized in the olfactory bulb, cortex, and hippocampus, with lower levels observed in other brain regions (Figure 1B). Given that PrP^C^ is widely expressed throughout the CNS, and in vitro studies on prion propagation have indicated that Nbs must bind to nearly all normal PrP to effectively inhibit PrP conversion [25], the confined regional expression of Nbs is likely insufficient to prevent the conversion of PrP^C^ to PrP^Sc^ during prion disease. Moreover, within the complex in vivo extracellular environment, Nbs will encounter conditions that differ significantly from those found in vitro, including factors such as clearance and degradation. This underscores the importance of conducting in vivo studies. Second, while transgene expression remained relatively stable for 180 days, it became unstable during the terminal stage of prion disease. Specifically, the levels of Nb196 and Nb484 were close to zero, with only Nb862 retaining a higher expression level (Figure 4A), which may account for the modest therapeutic effect observed in mice expressing Nb862.

To achieve a more substantial therapeutic effect, several enhancement strategies may be explored. First, since PrP^Sc^ is less abundant than PrP^C^ [6] and Nbs are known to prefer conformational epitopes [30], identifying Nbs that specifically target PrP^Sc^ would be ideal, which will also minimize potential impacts on the physiological function of PrP^C^ [29]. In addition, the Nbs used in our study are high-affinity binders to PrP^C^ [24,25], but it remains uncertain whether this binding is maintained in the complex in vivo membrane environment. Further screening for Nbs with high affinity for PrP^C^ on cell membranes will be another strategy for enhancement.

An interesting observation from our study is that all three anti-PrP^C^ Nbs appeared to slow bodyweight loss during the clinical phase of prion disease (Figure 3B), suggesting an attenuation of the neurotoxic process. It is well established that in prion disease, PrP^Sc^ can induce changes in PrP^C^, with rapid neurotoxicity being executed by altered PrP^C^ rather than PrP^Sc^ [31,32]. It is possible that these PrP^C^-binding Nbs, although insufficient to inhibit the PrP^C^-to-PrP^Sc^ conversion in vivo due to expression levels and/or patterns, can bind to PrP^C^ and interfere with its neurotoxic activity. Further studies are necessary to determine whether these PrP^C^-binding Nbs could complement the currently most promising therapies against prion disease, specifically the reduction of PrP^C^ expression through antisense oligonucleotides [33], genome editing using targeted epigenetic approaches [34], or base editing strategies [35].

The ICV rAAV injection method enables us to evaluate the efficacy of an Nb in treating prion disease and serves as a proof-of-principle study. While this approach is suitable for the current stage of our study, it is impractical for real-world applications. Intranasal or intravenous injections are more viable alternatives, which also allow for repeated dosing to maintain high transgene levels at different stages of prion disease. For intravenous administration, selecting AAV serotypes capable of crossing the BBB is critical. Rapid advancements in this field of research [36] are likely to provide solutions to this challenge in the near future.

## 5. Conclusions

Our study establishes that ICV injection of rAAV in newborn mice is a feasible approach for evaluating the safety and efficacy of Nbs in vivo. We demonstrated that high-level expression of these three anti-PrP^C^ Nbs in the CNS is safe and can yield beneficial effects against prion disease. However, further improvements are necessary to achieve meaningful therapeutic outcomes. 

## Data Availability

All data are contained within the article. Further inquiries can be directed to the corresponding author.

## Abbreviations

The following abbreviations are used in this manuscript:

rAAV	Recombinant adeno-associated virus
ICV	Intracerebroventricular
BBB	Blood–brain barrier
Nbs	Nanobodies
CNS	Central nervous system
gc	Genome copy
ELISA	Enzyme-linked immunosorbent assay
WB	Western blotting
OB	Olfactory bulb
CTX	Cortex
HIP	Hippocampus
MB	Midbrain
BS	Brainstem
CERE	Cerebellum
PK	Proteinase K
HE	Hematoxylin-eosin
dpi	Days post-injection
